# Differences in RNA Binding Between Segmented and Non-Segmented Negative-Strand Virus Nucleocapsids

**DOI:** 10.3390/microorganisms14061194

**Published:** 2026-05-26

**Authors:** Rob W. H. Ruigrok, Allison Ballandras-Colas, Thibaut Crépin, Hélène Malet, Dan Kolakofsky

**Affiliations:** 1 University Grenoble Alpes, CNRS, CEA, IBS, F-38000 Grenoble, France; allison.ballandras-colas@ibs.fr (A.B.-C.); thibaut.crepin@ibs.fr (T.C.); helene.malet@ibs.fr (H.M.); 2 Department of Microbiology and Molecular Medicine, Faculty of Medicine, Medical School, University of Geneva, 1211 Geneva, Switzerland

**Keywords:** RNA, nucleocapsid, negative-strand RNA virus

## Abstract

Segmented and non-segmented negative-strand RNA viruses share the same general pathway for genome transcription, which generates messenger RNA, and genome replication which duplicates the viral RNA. These processes are performed by the viral polymerase and necessitate the viral RNA to be coated by a non-covalent polymer of nucleoproteins known as nucleocapsid. The non-segmented negative-strand RNA viruses (nsNSVs) have rigid nucleocapsids covering the entire tightly bound genome and require a phosphoprotein cofactor for proper replication and transcription by the polymerase, while the segmented negative-strand RNA viruses (sNSVs) have very flexible nucleocapsids with only few nucleotides tightly bound to each nucleoprotein, and their viral RNA genome ends are directly bound to the polymerase. We discuss here how the differences in RNA binding are likely to be crucial for proper replication and transcription in both nsNSVs and sNSVs.

## 1. Introduction

Upon entering a cell, negative-strand RNA virus (NSV; *Negarnaviricota*) replication obligatorily begins with the synthesis of viral mRNA from the viral genomic RNA (vRNA). These viral genomes are never present as free RNAs; they are always associated with multiple copies of the viral nucleoprotein (N/NP). N/NP specifically binds single-stranded RNA and oligomerizes into a non-covalent helical polymer that encapsidates the genomic and antigenomic RNAs. NSVs do not require RNA helicases, as the N/NP-RNA nucleocapsid prevents the formation of double-stranded RNA between viral mRNAs and the negative-sense genomic RNA. The N/NP shell also forms a protective barrier against antiviral host proteins, a function that is particularly important early in infection before newly synthesized viral proteins assemble viral replication factories.

NSVs are broadly divided into two groups. The first comprises viruses with a single, continuous RNA genome in which 5–10 genes are arranged sequentially, each flanked by transcriptional start and stop signals. These viruses are referred to as non-segmented NSVs (nsNSV) and belong primarily to the order *Mononegavirales*, which includes viruses such as measles virus, rabies virus, human respiratory syncytial virus (hRSV), Ebolavirus, and Borna disease virus. The second group comprises segmented NSVs (sNSV), whose genomes are divided into multiple RNA segments, each encoding one or more viral proteins. These viruses are classified in two classes named *Insthoviricetes* and *Bunyaviricetes*. *Insthoviricetes* contains the order *Articulavirales* that includes viruses such as influenza virus. *Bunyaviricetes* includes the *Elliovirales* and *Hareavirales* orders and encompasses emergent viruses such as Lassa virus, Crimean-Congo hemorrhagic fever virus, Rift Valley fever virus, and Hantaan virus. There are many reviews on the structures on NSV nucleocapsids [1,2,3,4,5,6] that mainly describe the nucleoproteins and their assemblages. This review, in contrast, treats how the viral RNA binds to the N/NP within the nucleocapsids.

## 2. RNA in Non-Segmented Viruses

**Figure 1:**
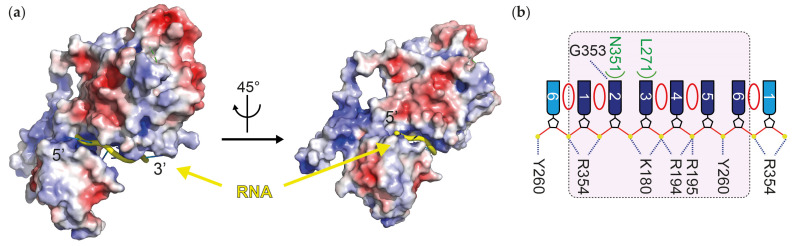
Measles virus nucleoprotein–RNA complex. (**a**). Structure of a single protomer of measles N-RNA (PDB code: 6H5Q); positive charges are in blue, and negative charges are in red. The RNA (shown as a yellow ribbon) is hardly visible in N surface representation, as it is within a narrow binding groove between the 2 lobes N. (**b**). Schematic representation of a nucleoprotein (pink box) RNA complex. The yellow and black colors represent the phosphate and ribose of the RNA backbone, respectively. Six RNA bases bound to the nucleoprotein are shown as dark blue rectangles and labelled 1 to 6. The RNA bases that are not bound to the protomer appear as blue rectangles. The red circles, the blue dotted lines, and the green semicircles indicate the stacked nucleobases, the hydrogen bonds, and the hydrophobic interactions, respectively. Nucleotides 6-1-2 are stacked (indicated by red circles), and the bases are pointing away from the protein; bases 3-4-5 stack as well and point towards the nucleoprotein. All phosphates are bound to the protein.

The RNA of non-segmented negative-strand RNA virus (nsNSVs) cannot easily be cut by RNases [7,8,9] because all phosphates of the backbone are bound to the nucleoprotein (Figure 1b). The nucleocapsids are rather rigid structures. Their protomers have masses between 41 and 83 kDa (Table 1), and the nucleoprotein weight per nucleotide is between 5 and 14 kDa (the measles virus nucleoprotein has a mass of 58,131 Da and is associated with six nucleotides per protomer, therefore, 9688 protein Da per nucleotide; see Table 1). nsNSV nucleoproteins have two domains, with a narrow and positive-charged cleft between them in which the genome RNA is bound (Figure 1a). nsNSV nucleoproteins bind a fixed number of nucleotides per protomer, between six to nine nts among different families. The RNA cleft can bind six nucleotides per protomer for paramyxoviruses, like measles [10], mumps [11], or murine respirovirus [12,13], and for filoviruses, like Ebola [14,15,16] or Marburg [17]. The nucleoprotein of pneumovirus (hRSV and hMPV) binds seven nucleotides [18,19], the protomer of Borna disease virus 1 binds eight nucleotides [20], and the nucleoprotein for rabies and vesicular stomatitis virus (VSV) binds nine nucleotides [21,22]. The nucleotide bases can point either toward or away from the protein core and either stack with each other or with amino acid residues [23] (Figure 1b). The nucleotide stacks make A-form structures, and their direction changes every three or four nucleobases. Therefore, unless the viral polymerase is actively engaged in RNA synthesis, the nucleotides bound within the nucleoprotein are as structurally constrained as the nucleocapsid itself in *Mononegavirales* [6].

## 3. RNA in Segmented Viruses

The nucleocapsids of segmented negative-strand RNA virus (sNSVs) are very different and very flexible [3,6]. Their vRNA is not entirely bound to nucleoproteins, as the viral polymerases bind both ~10 terminal nucleotides of the 5′ and 3′ termini into two separate and specific single-stranded RNA binding sites, preventing hybridization of these nucleotides despite their complete complementarity. Nucleotides ~10 to 20 of the 5′ and 3′ end form a distal duplex that is essential for the subsequent initiation of replication and/or transcription. The remaining genome RNA, which is associated with nucleoproteins, forms the nucleocapsid that can be easily cut by RNAse treatment *in vitro* [36,37,38].

A notable shared property of Bunyaviruses and Articulaviruses nucleocapsids is their pronounced flexibility, which prevents their high-resolution (∼3 Å resolution) X-ray or cryo-EM structures’ determination and therefore impairs the understanding of the molecular interaction between long viral RNA and nucleoproteins. Instead, only high-resolution structures of N/NP-RNA rings with a defined number of N/NP proteins and limited-length RNA have been obtained. These *in vitro* assemblies, while informative for protein-RNA interactions, do not fully represent nucleocapsids due to their restricted size. To obtain molecular insight into binding of longer RNA to sNSV nucleocapsids, likely to reflect native nucleocapsid organization, N/NP-RNA nucleocapsid-like complexes have been reconstituted for influenza A and Hantaan virus [26,34], which are more likely to reflect native nucleocapsid organization. In addition, a mini-RNP containing nine NPs and one polymerase, both bound to RNA, has been structurally determined for influenza [35]. Many of these cryo-EM or X-ray structures were generated *in vitro* using defined RNA sequences, such as poly-U for Rift Valley fever virus or poly-UC for influenza structures. The Hantaan nucleocapsid-like structure was formed during the nucleoprotein expression in insect cells and is therefore bound to endogenous RNA in an aspecific manner.

**Figure 2:**
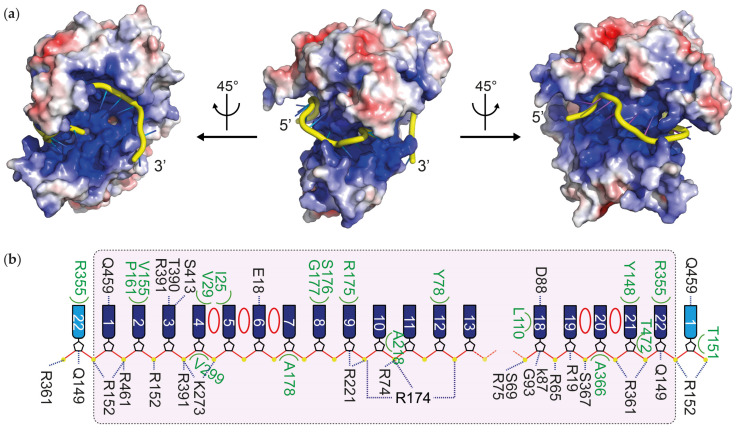
RNA binding by influenza virus nucleoprotein. (**a**). Single protomer of the influenza N-RNA in the RNP-like structure (PDB code: 9GAS); positive charges are in blue, and negative charges are in red. The RNA is shown as a yellow ribbon. The structure shows a large positive area for RNA binding. (**b**). Schematic representation of Strand 2 in the RNP-like structure (ref. [34]). The light pink box represents the considered protomer of nucleoprotein. The yellow and black colors represent the phosphate and ribose of the RNA backbone, respectively. The red circles, the blue dotted lines, and the green semicircles indicate the stacked nucleobases, the hydrogen bonds, and the hydrophobic interactions, respectively. Most phosphates are bound to NP, except nucleotides 4 to 7, where NP binding occurs through base stacking rather than interactions with the phosphate groups. N.B. For this figure, we have changed the numbers of the nucleotides from the original figure in [34].

Except influenza A nucleoprotein, which is comparable in size, most sNSV nucleoproteins are smaller than those of nsNSVs. However, they bind lower protein mass per bound nucleotide, ranging from 2.4 to 4 kDa per nucleotide (Table 1). In addition, their RNA-binding cleft is significantly larger (see Figure 1a versus Figure 2a), indicating a greater capacity for RNA interaction compared to the narrower cleft observed in nsNSVs.

Presently, there are 11 high-resolution N/NP-RNA structures showing that sNSV nucleoproteins can accommodate a broader range of nucleotides per protomer (Table 1). From the class *Bunyaviricetes*, the two Phenuiviruses (Order *Hareavirales*), Rift Valley fever virus (RVFV) and Toscana virus, accommodate between six and seven bases per N protomer [31,32], and the four peribunyavirus nucleocapsid structures (Order *Elliovirales*) have 11 bases per protomer [23,27,28,29]. With the four latter, nucleotides 9, 10, and 11 make minimal contact with the nucleoprotein. The large nucleoprotein of Lassa virus (Order *Hareavirales*) comprises 569 residues, featuring an N-terminal domain (residues 1–340) similar to those of other bunyavirus nucleoproteins and a C-terminal ExoN domain that degrades double-stranded RNA [39]. The N-terminal domain binds eight RNA bases, and the structure of the N-RNA shows a narrow RNA path only for residues 2–4 [30], but residues 5–8 are more flexible. Similarly, the cryo-EM structure of the RNA-bound nucleocapsid of Hantaan virus (Order *Elliovirales*) reveals that the nucleoproteins form a long, tightly wound nucleocapsid helix containing a continuous positively charged groove likely to be able to accommodate long RNA [26]. Only three bases are resolved per protomer; the remaining nucleotides are likely flexible and not stably associated with the nucleoprotein, which would explain why they are not visible in the cryo-EM structure.

Within the order *Articulavirales*, the nucleoprotein of tilapia lake virus (family *Amnoonviridae*) binds 12 nucleotides [33]. In the N-RNA structure of the pseudo-C5 oligomer, each protomer accommodates 12 nucleotides; however, nucleotides C2 to A4 do not interact with the protein. Instead, their bases stack together and remain flexible. There are several nucleocapsid-like structures of influenza A virus (family *Orthomyxoviridae*) with up to 18 bases, but there is place for more nucleotides, up to 24 bases per protomer [34,40,41]. More recently, the structure of the influenza A virus mini-RNP [35], originally designed by the group of Juan Ortín [42], showed that influenza nucleoprotein can bind 24 nucleotides.

In influenza-like nucleocapsids, four structures have been determined using an N-terminally truncated nucleoprotein (residues 15–498) in complex with poly-UC RNAs of varying lengths (12, 14, and 18 nucleotides; Figure 2). In all cases, the 5′ end of the RNA is positioned between two NP protomers, and the first seven nucleotides adopt a highly conserved conformation, reminiscent of the RNA binding observed in orthobunyavirus N-RNA complexes. Beyond this conserved region, the RNA-binding groove becomes wider, allowing downstream nucleotides to adopt multiple conformations. In the double antiparallel assembly containing an 18-nucleotide RNA, two distinct trajectories are observed: a relaxed strand (strand 1) and a stretched strand (strand 2) ([34]; Figure 2b). In strand 2, nucleotides 5–8 do not interact with the protein and instead form a stacked arrangement of bases, similar to nucleotides 2–4 in the tilapia lake virus nucleoprotein-RNA structure [33]. Notably, the NP-RNA complex within the mini-RNP adopts a particularly open conformation, with only 10 nucleotides resolved in contact with the protein [35]. This observation suggests that the remaining nucleotides are flexible and likely not stably associated with the nucleoprotein.

From the structures of the segmented viruses mentioned here, one could see that the RNA phosphate–ribose backbone is often not bound to the nucleoprotein and, therefore, is sensitive to RNases.

## 4. Hypothesis: The Flexible Nucleotides of the Segmented Viruses Can Slide on the Nucleoprotein Inside the Viral Capsids

Nucleocapsids of segmented NSV need to protect the viral RNA while supporting the complex processes of vRNA replication/transcription and the formation of a nascent nucleocapsid. We hypothesize four criteria to be essential for these processes: (i) the flexibility of the nucleocapsid, (ii) the tight binding of only few nucleotides per nucleoprotein, (iii) the strong interaction between adjacent nucleoproteins, and (iv) the loose interactions between more distant nucleoproteins. Indeed, during replication and transcription, the polymerase needs to transiently access to the RNA without disrupting the overall architecture of the nucleocapsid. For this, a working model hypothesis is that the viral RNA that enters the polymerase would detach from few nucleoproteins of the nucleocapsid without perturbing the interactions between adjacent nucleoproteins that transiently become RNA-free. After being copied by the polymerase, the viral RNA would then bind again to the same nucleoproteins. For this to happen easily, a flexible nucleocapsid with few nucleotides tightly bound and strong interactions between adjacent nucleoproteins appears ideal to favor transient RNA release from the nucleocapsid during copying, which would bind again when exiting the polymerase.

The same logic could be applied for the proper relative positioning of the eight RNPs during influenza virion formation. This step follows the exit of influenza virus RNP from the nucleus to form the complete genome that buds from the cell. This remarkable eight-part assembly [43,44] is thought to result from stem-loop RNA structures that extrude from each of the various nucleocapsid segments and interact with similar structures from other RNPs to form the complete eight RNA-segment genome [45,46]. It is unlikely that these extruded RNAs are present all the time because they could be sensitive to RNases or bind cell proteins that induce an immune response. It is more likely that these extruded RNAs would be in dynamic equilibrium with alternate structures on each nucleocapsid that form and change during the life cycle of each segment. This interconversion of alternate RNA:nucleoprotein interactions on each segment would be greatly aided by the ability of the RNA to detach and reattach easily to the nucleoprotein. Here again, having only few nucleotides tightly bound would ensure an easy transient extrusion of the RNA.

Having few nucleotides tightly bound, strong adjacent nucleoprotein interactions, and loose interactions between more distant nucleoproteins is also likely to be essential for nascent RNP formation in influenza. One could indeed hypothesize that the addition of a new nucleoprotein onto a nascent, right-handed-growing, and anti-parallel nucleocapsid in influenza is required to modify the relative positioning of the non-adjacent nucleoproteins, while keeping intact the interactions between adjacent nucleoproteins. Tight binding of a few nucleotides per nucleoprotein would prevent sliding of the vRNA and might be essential to preserve nucleocapsid integrity. The loose binding of the other nucleotides to the nucleoprotein would be necessary to enhance the necessary flexibility of this highly dynamic process.

Non-segmented NSV strongly differ from segmented NSV, as the entire RNA backbone of the nsNSV nucleocapsids is bound to the nucleoprotein polymer. Moreover, given the changes in direction of the A-form structures (whose bases point as a group toward or away from the nucleoprotein core), the nucleotides cannot be detached from the protein without extra energy. This tight binding of the RNA allows the replication of paramyxoviruses to be governed by “the rule of six”; only genomes whose total lengths are precisely a multiple of six nucleotides are viable. The ability of the polymerase to access the RNA in this tight configuration would not be possible without the action of the phosphoprotein P that appears as a central actor for disruption of nucleoprotein interactions and exposure of the RNA for its replication.

Overall, the flexible and dynamic nature of sNSV nucleocapsids, characterized by partial RNA binding, strong local protein interactions, and the ability of nucleoproteins to slide along the RNA, appears to be a key evolutionary adaptation. This structural plasticity supports efficient replication, precise genome assembly, and regulated RNA accessibility, distinguishing segmented viruses from their more rigid non-segmented counterparts.

## Figures and Tables

**Table 1:** RNA–nucleoprotein structures of the negative-strand RNA viruses.

Virus	N/NP (Residues)	Mass (Da)	Nucleotides per Protomer (nt)	Mass Protein/nt (Da)	References
**Non-segmented Negative-strand RNA Virus**					
Phylum *Negarnaviricota*, Subphylum *Haploviricotina*, Class *Monjiviricetes*, Order *Mononegavirales*					
Family *Bornaviridae*					
Borna disease virus 1	372	40,931	8	5116	[20]
Family *Filoviridae*					
Ebola virus	739	83,300	6	13,883	[14]
Marburg virus	695	77,747	6	12,958	[17]
Family *Paramyxoviridae*					
Measles virus	525	58,131	6	9688	[10]
Mumps virus	549	61,366	6	10,228	[11]
Avian orthoavulavirus 1 ^1^	489	53,023	6	8835	[24]
Nipah virus	532	58,168	6	9694	[25]
Murine respirovirus ^2^	517	56,762	6	9460	[12]
Family *Pneumoviridae*					
hRSV	391	43,493	7	6213	[18]
hMPV	394	43,538	7	6620	[19]
Family *Rhabdoviridae*					
Rabies virus	450	50,605	9	5627	[21]
VSV	422	47,368	9	5263	[22]
**Segmented Negative-strand RNA Virus**					
Phylum *Negarnaviricota*, Subphylum *Polyploviricotina*, Class *Bunyaviricetes*, Order *Elliovirales*					
Family *Hantaviridae*					
Hantaan virus ^3^	429	48,142	3 ^3^	NC ^5^	[26]
Family *Peribunyaviridae*					
Leanyer virus	235	26,259	11	2387	[27]
Bunyamwera virus	233	26,664	11	2424	[28]
La Crosse virus	235	26,530	11	2412	[29]
Schmallenberg virus	233	26,181	11	2380	[23]
Phylum *Negarnaviricota*, Subphylum *Polyploviricotina*, Class *Bunyaviricetes*, Order *Hareavirales*					
Family *Arenaviridae*					
Lassa virus ^4^	1–340	38,200	8	4775	[30]
Family *Phenuiviridae*					
Rift Valley fever virus	245	27,360	6–7	3909–4560	[31]
Toscana virus	253	27,704	7	3958	[32]
Phylum *Negarnaviricota*, Subphylum *Polyploviricotina*, Class *Insthoviricetes*, Order *Articulavirales*					
Family *Amnoonviridae*					
Tilapia lake virus	354	38,400	12	3200	[33]
Family *Orthomyxoviridae*					
Influenza A virus	498	56,210	24	2342	[34,35]

^1^ New name for Newcastle disease virus. ^2^ New name for Sendai virus. ^3^ Only 3 nucleotides are visible in the cryo-EM structure, but the number of bases per N is unknown. ^4^ N-terminal domain. ^5^ not calculated.

## Data Availability

The original contributions presented in this study are included in the article. Further inquiries can be directed to the corresponding authors.

## Abbreviations

The following abbreviations are used in this manuscript:

vRNA	viral genomic RNA
nsNSV	non-segmented negative-strand RNA virus
sNSV	segmented negative-strand RNA virus
N/NP	nucleoprotein
